# Morphological and behavioural changes occur following the X-ray irradiation of the adult mouse olfactory neuroepithelium

**DOI:** 10.1186/1471-2202-13-134

**Published:** 2012-10-31

**Authors:** Carla Cunha, Yvonne Hort, John Shine, Kharen L Doyle

**Affiliations:** 1 Neuroscience Research Program, Garvan Institute of Medical Research, Sydney, Australia

**Keywords:** Olfactory neuroepithelium, X-ray irradiation, Proliferation, Apoptosis, Adult stem cells

## Abstract

**Background:**

The olfactory neuroepithelium lines the upper nasal cavity and is in direct contact with the external environment and the olfactory bulbs. The ability to self-renew throughout life and the reproducible recovery after injury, make it a model tissue to study mechanisms underlying neurogenesis. In this study, X-rays were used to disrupt proliferating olfactory stem cell populations and to assess their role in the cellular and morphological changes involved in olfactory neurogenic processes.

**Results:**

We have analysed the histological and functional effects of a sub-lethal dose of X-rays on the adult mouse olfactory neuroepithelium at 2 hours, 24 hours, 1 week, 2 weeks and 5 weeks. We have shown an immediate cessation of proliferating olfactory stem cells as shown by BrdU, Ki67 and pH3 expression. At 24 hours there was an increase in the neural transcription factors Mash1 and Pax6 expression, and a disruption of the basal lamina and increase in glandular cell marker expression at 1 week post-irradiation. Coincident with these changes was an impairment of the olfactory function *in vivo*.

**Conclusions:**

We have shown significant changes in basal cell proliferation as well as morphological changes in the olfactory neuroepithelium following X-ray irradiation. There is involvement of the basal lamina as well as a clear role for glandular and sustentacular cells.

## Background

The olfactory neuroepithelium (ON) is a specialized epithelium responsible for the perception of odours and undergoes neurogenesis throughout life in order to cope with damage suffered by being in direct contact with the external environment. It is also an easily accessible source of stem cells for cell therapy, so that the ON has become a useful model of neurogenesis [1,2].

The ON is a simple pseudostratified epithelium composed, in a basal to apical organization, of horizontal basal cells, globose basal cells, olfactory sensory neurons (OSN), Bowman's gland duct cells, microvillar cells and sustentacular cells. Underlining the ON, the lamina propria is composed of connective tissue, OSN axon bundles and respective olfactory ensheathing glia, blood vessels and Bowman's glands [3,4]. Many studies have involved the identification of the ON stem cell and both horizontal basal cells and globose basal cells have been identified as two distinct putative stem cell populations, responsible for giving rise to all the different cell types within the ON and having different roles in normal neurogenesis and neuronal replacement after injury [1,5-9]. In previous studies sustentacular cell endfeet showed expression of the neural stem cell marker, nestin [10] and were likened to radial glial cells. Isolated populations of glandular cells were able to proliferate and form olfactory neurospheres [11]. This suggests that there are other potential populations of olfactory stem cells within the mouse ON.

Different models of ON degeneration have been used, including the exposure to the solvents zinc sulphate [12,13], acetone [14], toluene [15] and the anti-thyroid drug, methimazole [16,17]. Another approach has been the inhalation of volatile chemicals, such as CO_2_[18] and methyl bromide [19,20]. All of these methods rely on a widespread variable degeneration of the ON, destroying multiple cell populations, without specifically targeting olfactory stem cells. Bulbectomy instead induces a retrograde degeneration of OSNs but does not allow for axonal re-targeting to the olfactory bulb [21,22].

In this study, we analysed the effects on neuronal and non-neuronal cells within the ON following X-ray irradiation of proliferating olfactory stem cells. Neural stem cells are more vulnerable than other cell types to ionizing radiation. It has been shown that ionizing radiation induces apoptosis of proliferating stem cells in the dentate gyrus of the adult rat hippocampus [23]. X-ray irradiation of young adult rat brain has resulted in apoptosis especially in the subependyma, a region harbouring a population of proliferating neural and glial stem cells [24-26]. Moreover, it has been demonstrated that X-ray irradiation can specifically target the neural stem cell population in the rat brain subventricular zone [27].

We hypothesize, based upon previous work published by our group, that glandular cells, apically-situated sustentacular cells as well as suspected basal cells will be affected by X-ray irradiation and cause both morphological and behavioural disruption of the mouse ON. This study contributes to the understanding of cellular events that occur within several olfactory cell populations after an induced selective damage to the proliferating olfactory stem cell populations.

## Results

**Figure 1:**
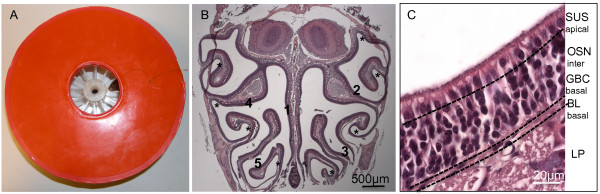
**X-ray irradiation of mice and morphological analysis.A**, X-ray irradiation was performed on anaesthetized adult mice placed in a circular Perspex pie chamber, covered with a lead shield (red) to protect the whole body except the nose snout. The OB were protected from irradiation, leaving the ON exposed. B,C, Photomicrographs showed the anatomy of the mouse ON stained with H&E. **B**, Coronal section/anteroposterior section of the ON showing the approximate location of the 5 areas analyzed for all immunohistochemical analysis, corresponding to the middle of the nasal septum (1), one more superior and one more inferior area of both the left (2, 3) and right (4, 5) turbinates. **C**, Higher magnification of the ON at the level of the turbinates, showing the basal lamina (BL) in the basal layer, the sustentacular cell nuclei layer (SUS) in the apical layer, the olfactory sensory neurons (OSN) in the intermediate layer and globose basal cells (GBC) also in the basal layer, and the underlying lamina propria (LP). * Bowl-like areas.

Anaesthetized adult mice were irradiated with a sub-lethal dose of X-rays on the mouse ON. The olfactory bulbs (OB) were protected from irradiation by use of a lead shield designed to protect the whole body except the nose snout (Figure 1A). Experimental groups were as follows: unlesioned control group, 2 hours, 24 hours, 1 week, 2 weeks and 5 weeks post-X-ray irradiation groups, with all animals injected with BrdU 2 hours before perfusion. Mice were monitored and weighed daily. None of the irradiated mice presented with oral or nasal ulceration, nasal discharge, breathing problems, nose scratching or signs of distress at any level. Olfactory behavioural tests were performed as well as ON detailed immunohistochemical analysis. Due to the complexity and timing of the olfactory behavioural tests, these were not performed for the 2 hours group.

### Morphology of the ON and OB post-irradiation

Haematoxylin and eosin (H&E) staining was performed on both ON (including the vomeronasal organ) and OB and sections analyzed at roughly the same coronal level for each group. For the ON, 5 areas (4 within the turbinates and 1 within the nasal septum) were defined for morphological analysis and for posterior detailed immunohistochemical analysis (Figure 1B). The gross morphology of the turbinates and nasal septum as discerned using low magnification microscopy was identical for all groups indicating that following irradiation no gross abnormalities occurred within the mouse ON (Figure 1B,C) or vomeronasal organ (results not shown).

**Figure 2:**
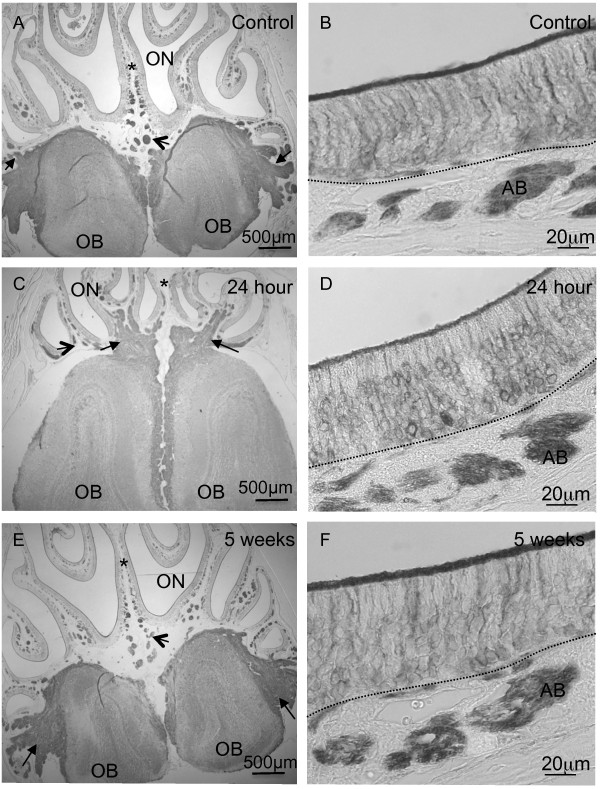
**Morphology of ON and OB following X-ray irradiation. A,C,E,** Photomicrographs of olfactory turbinates (including the OB and ON) labelled with the βIIITubulin monoclonal antibody. **A**, In the control animals at low magnification, the axon bundles were labelled positively with βIIITubulin (open arrow) and positively labelled nerve fibres were seen surrounding the olfactory bulbs (black arrows). **C**, 24 hours post-irradiation axon bundles remained βIIITubulin-positive (open arrow) and βIIITubulin-positive nerve fibres (black arrows) were seen linking the ON with the OB which is the same as at 5 weeks post-irradiation (**E**). **B**,**D**,**F**, Photomicrographs of the ON at higher magnification labelled with βIIITubulin. **B**, The cell soma of OSNs, their processes, cilia and axon bundles were βIIITubulin-positive in the control mice. **D**,**F**, 24 hours and 5 weeks post-irradiation the distribution of βIIITubulin was the same as in the control mice. The dashed line in **B**,**D**,**F** represents the basal lamina. AB, axon bundle. (n=3 mice per group). * indicates septum.

We analysed the morphology of the ON, OB as well as the nerve fibres connecting them. We studied these structures following staining of βIIITubulin. We showed that in the controls and at all time points following irradiation (only 24 hour and 5 week post-irradiation in figure) βIIITubulin was present in the cell soma of OSNs, processes and axon bundles in the ON (Figure 2B,D,F). Furthermore, at low magnification the βIIITubulin-positive nerve fibres can be seen traversing from the ON to the OB in the control as well as the 24 hour and 5 week groups (Figure 2A,C,E). Higher magnification of the immunohistochemical analysis of βIIITubulin in the OB can be seen in Additional file 1. Qualitative analysis reveals there was no differential localization in controls or irradiated mice. βIIITubulin labels the cell soma of glomerular and mitral cells as well as the nerve fibres in the untreated and treated mice.

**Figure 3:**
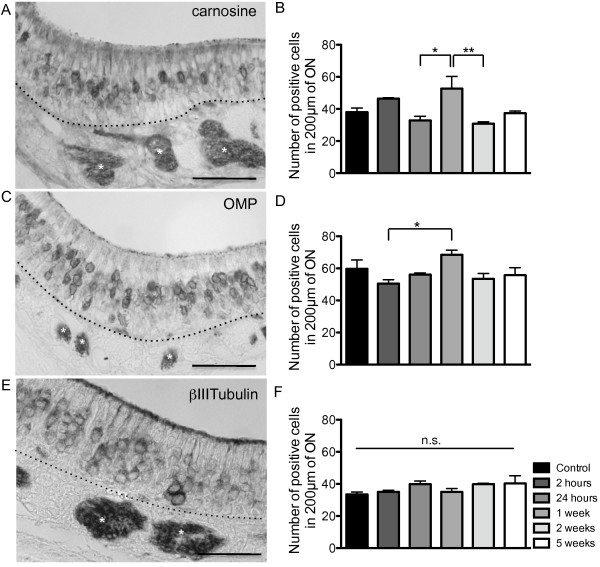
**Analysis of olfactory neurons in the ON at different time points post X-ray irradiation.A**,**B**, localisation and quantification of carnosine-positive neurons (n=3 mice); **C**,**D**, OMP-positive neurons (n=3 mice) and **E**,**F**, βIIITubulin-positive neurons (n=3 mice). (Carnosine: F_(5,12)_ = 5.765, p = 0.0061; OMP:F_(5,12)_ = 3.079, p = 0.0513). * p ≤ 0.05, ** p ≤ 0.01, n.s. not significant. **A**, carnosine, **C**, OMP and **E**, βIIITubulin labelling in the naive control group. (* white) Axon bundle. Dashed line indicates basal lamina. Scale bars = 50 μm.

We quantified the number of OSNs positively stained for βIIITubulin (immature and some mature OSNs) [28], carnosine (immature and mature OSNs) [10,11,29] and olfactory marker protein (OMP) (mature OSNs) [30,31] for all groups. The results showed no significant difference for βIIITubulin (Figure 3E,F), but there is an increase in the number of both carnosine- and OMP-positive OSNs at 1 week (Figure 3A-D) in the regions examined. Carnosine and OMP label more mature OSNs compared to βIIITubulin which labels only a subpopulation of mature OSNs [28].

### Proliferation was abolished immediately following irradiation

**Figure 4:**
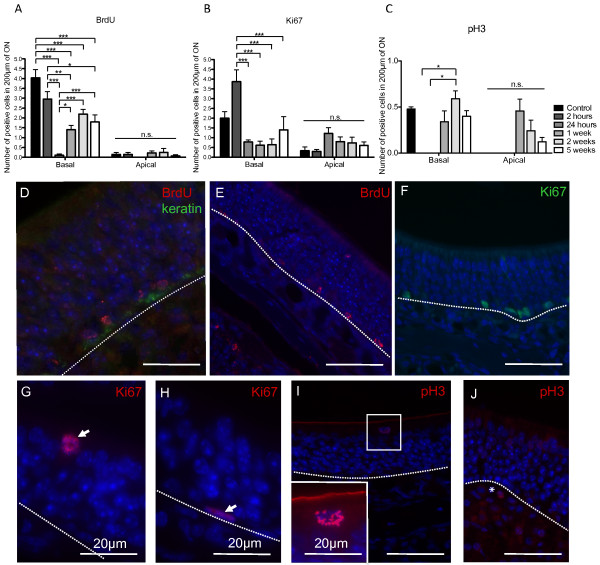
**Analysis of cell cycle markers in the mouse ON following X-ray irradiation.A**,**B**,**C**, Proliferation in the ON for different time points following X-ray irradiation, as quantified by **A**, BrdU incorporation (F_(11,78)_ = 28.02, p ≤ 0.0001, n=3 mice); **B**, Ki67 expression (F_(11,40)_ = 7.622, p ≤ 0.0001, n=3 mice); **C**, pH3 expression (F _(11,40)_ = 4.369, p = 0.0003, n=3 mice). * p ≤ 0.05, ** p ≤ 0.01, *** p ≤ 0.001, n.s. not significant. **D**,**E**, BrdU was mainly observed on cells lying on top of the basal lamina (keratin). Ki67 expression at 2 hours in the basal lamina (**F**), in the sustentacular cell layer at 1 week (arrow) (**G**) and a horizontal basal cell positive for Ki67 at 2 weeks (arrow) (**H**), pH3 expression in the sustentacular cell layer at 5 weeks (**I**) and in the lamina propria at 2 weeks (**J** *). Dashed lines indicate basal lamina. All sections have nuclei counterstained with DAPI (blue). Scale bars: **D-F**,**I**,**J**, 50 μm.

For analysis of cell proliferation, three different markers were analyzed: detection of BrdU incorporation during the S phase of the cell cycle (Figure 4A,D,E); Ki67, a marker of cell proliferation at G1, S and G2 phases of the cell cycle (Figure 4B,F-H) and pH3, a marker of mitosis (Figure 4C,I,J). BrdU staining showed a significant reduction in the number of proliferating cells at the level of the basal cell layer, which decreased at 2 hours and was almost abolished at 24 hours. The number of BrdU-positive cells returned to half the control level after 1 week and remained so up to 5 weeks (Figure 4A). There was a small proportion of BrdU-positive cells within the sustentacular cell layer, which disappeared at 24 hours (Figure 4A). BrdU and keratin, a marker for horizontal basal cells, did not generally co-localize, with most of the BrdU-positive cells appearing on top of keratin-positive cells in the globose basal cell layer (Figure 4D). Still, we did observe some cells positive for both BrdU and keratin at 1 week, indicating that a small percentage of proliferating cells were indeed horizontal basal cells at least at 1 week post-irradiation (results not shown).

We found an increase in Ki67-positive cells in the basal layer of the ON at 2 hours, which was significantly reduced below normal levels after 24 hours (Figure 4B). Figure 4G,H shows a sustentacular cell and a horizontal basal cell labeled with Ki67, respectively. Overall there were more Ki67-positive cells in the sustentacular cell (apical) layer (Figure 4B).

In order to more clearly dissect cell proliferation within the ON, we have analyzed also pH3, a specific marker for mitosis. Results are in agreement with BrdU, in that we have found mitosis at the level of the basal lamina, showing a reduction at 2 hours and 24 hours, with values returning almost to normal after 1 week (Figure 4C). Positive cells were seen also at the sustentacular cell layer at 1, 2 and 5 weeks (Figure 4C,I), indicating an increase in mitosis in both the basal and apical layers following irradiation. There were also a few pH3-positive cells within the lamina propria at 2 weeks (Figure 4J).

The overall analysis of proliferation markers shows a significant reduction in olfactory progenitor cell proliferation following X-ray irradiation, which is almost abolished after 24 hours, and returning to normal levels after 5 weeks.

### X-ray irradiation did not induce apoptosis in the ON

**Figure 5:**
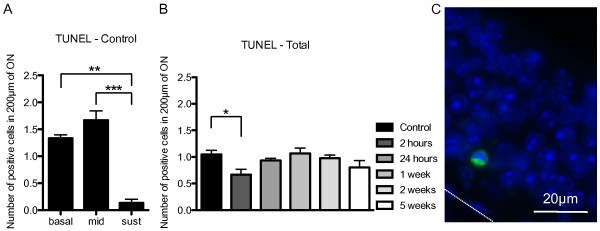
**Analysis of apoptosis in the mouse ON following X-ray irradiation.A**, Quantification of apoptotic cell nuclei in the ON of control naive animals in the basal cell layer, intermediate cell layers and sustentacular cell layer (F_(17,36) _= 8.804, p ≤ 0.0001, n=3 mice). **B**, Total number of apoptotic cell nuclei throughout the ON for each group (F_(5,12)_ = 2.862, p = 0.0629, n=3 mice). **C**, TUNEL labeling (green), DAPI (blue), 2 weeks post X-ray irradiation. Dashed line indicates basal lamina. * p ≤ 0.05,** p ≤ 0.01, *** p≤0.001.

Apoptotic cells were quantified by terminal deoxynucleotidyl transferase mediated dUTP nick end labeling (TUNEL), which measured fragmented DNA by incorporating fluorescein-12-dUTP at 3'-OH DNA ends, using the terminal deoxynucleotidyl transferase enzyme. Apoptotic cells were quantified separately at the level of the basal, intermediate and sustentacular cell layers and we saw a significantly higher number of apoptotic cells within the basal and intermediate cell layers of the ON (Figure 5A,C). This distribution was roughly the same for irradiated and non-irradiated groups. When the total number of apoptotic nuclei throughout the ON was quantified, we did not observe an increase in apoptosis. Instead, we observed a significant decrease in apoptosis 2 hours post-irradiation (Figure 5B).

### Anatomical morphology of the basal lamina post-irradiation

**Figure 6:**
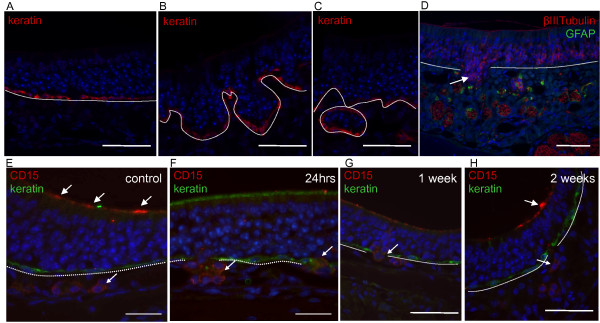
**Anatomical analysis of the mouse ON following X-ray irradiation.A-C**, keratin staining showing **A**, intact basal lamina in control mice and **B**,**C**, disrupted basal lamina at 1 week post X-ray irradiation. Keratin (red), DAPI (blue). **E-H**, CD15 staining post X-ray irradiation of the ON. **E**, in control mice keratin (green) labeled horizontal basal cells and CD15 (red) labeled glandular cells in the lamina propria (small arrow) as well as the apical mucous layer (white arrows). **F**, 24 hours post-irradiation CD15-positive glandular cells (small arrows) were seen in the lamina propria but no longer in the apical mucous layer. **G**, 1 week post-irradiation CD15-positive glandular cells were seen traversing the basal lamina (small arrow). **H**, 2 weeks following irradiation CD15 labeling returned to the apical mucous layer (white arrow) and CD15-positive cells remained in the lamina propria (small arrow). **D**, βIIITubulin-positive olfactory neurons were seen in a disruption of the basal lamina 5 weeks post X-ray irradiation (white arrow). GFAP-positive olfactory ensheathing glia were seen surrounding axon bundles. βIIITubulin (red), GFAP (green), DAPI (blue). Dashed line indicates basal lamina. Scale bars: **A-H**, 50 μm.

We noticed a significant disruption of the basal lamina starting 1 week post-irradiation, as seen by keratin-positive horizontal basal cells that line the basal lamina (Figure 6B,C), compared to the controls (Figure 6A). These areas did not present an increase in BrdU incorporation, showing there was no increase in proliferation where the basal lamina was disrupted (results not shown). When analyzing the position of the OSNs as indicated by βIIITubulin staining the basal lamina was not disrupted at 24 hours but was disrupted at 5 weeks where OSNs can be seen below the basal lamina in a pouch-like configuration within the lamina propria where βIIITubulin-positive axon bundles (red) and GFAP-positive ensheathing cells (green) are evident (Figure 6D).

### Migration of glandular cells post-irradiation

CD15 labels duct cells of the Bowman's gland in the ON and acinus cells in the lamina propria (Figure 6E-H). Importantly, CD15 expression was seen apically in the mucous layer of the ON (Figure 6E). At 24 hours CD15 expression was absent from the mucosal layer (Figure 6F), faint at 1 week (Figure 6G) and returning to normal levels 2 weeks following irradiation (Figure 6H). This result indicates involvement of Bowman's glands following irradiation.

Moreover we saw many CD15-positive cells in between the lamina propria and the ON where they do not normally appear (Figure 6F,G). Not all areas where the basal lamina was disrupted had CD15-positive cells and not all the cells in a single disrupted region were CD15-positive.

### Expression of neuronal transcription factors post-irradiation

**Figure 7:**
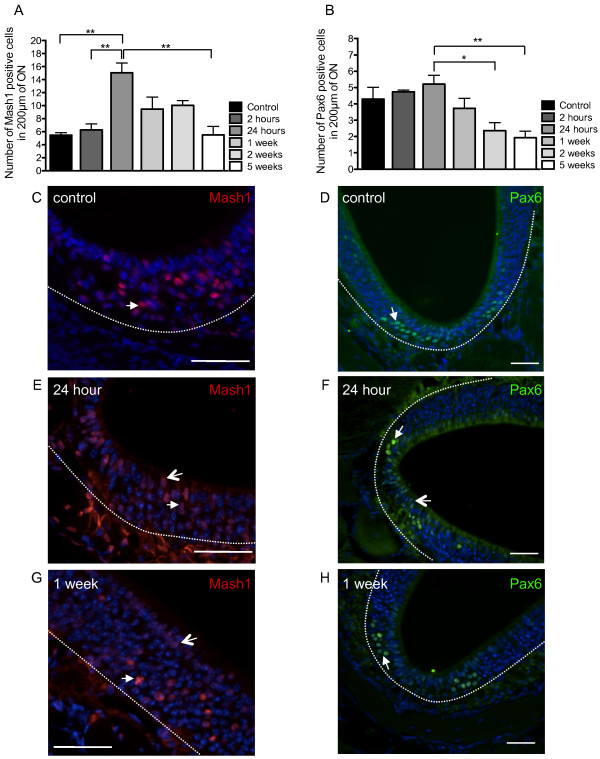
**Analysis of the transcription factors Mash1 and Pax6 following X-ray irradiation.** The quantification of Mash1 (**A**) (n=5 mice) and Pax6 (**B**) (n=5 mice) expression in the ON, for different time points post X-ray irradiation, showed a peak at 24 hours for both transcription factors (Mash1:F_(5,12)_ = 9.239, p = 0.0008; Pax6:F_(5,16 )_= 4.9991, p = 0.0061). Mash1 staining in **C**, control, **E**, 24 hours and **G**, 1 week post X-ray irradiation. There was nuclear expression of Mash1 in OSNs (white arrows) and sustentacular cells (open arrow). Pax6 staining in **D**, control, **F**, 24 hours and **H**, 1 week post X-ray irradiation. Pax6 localisation appeared in the nuclei of OSNs (white arrows) in controls (**D**), and 24 hours (**F**) and 1 week (**H**) post-irradiation. In particular, 24 hours post-irradiation, Pax6-positive nuclei appeared in the sustentacular cell layer (open arrow). Most of the Pax6 immunoreactivity appeared in the bowl-like areas of the ON. Dashed line indicates basal lamina. All sections have nuclei counterstained with DAPI (blue). Scale bars: C,E,G, 50 μm; D,F,H, 30 μm. * p ≤ 0.05, ** p ≤ 0.01.

We observed a statistically significant increase in both Mash1 (Figure 7A) and Pax6 (Figure 7B) expression in the mouse ON following irradiation, with a peak at 24 hours. Mash1 expression was seen in the nuclei of sustentacular cells and OSNs throughout the ON (Figure 7C,E,G). Pax6 nuclear expression was seen throughout the ON in the basal layer as well as the neuronal layer and the sustentacular cell layer (Figure 7D,F,H). The areas of the ON where Pax6 expression patterns were effected by irradiation was most evident in the bowl-like areas (Figure 1B, 7D, F, H). At 24 hours post-irradiation where there was an increase in expression of Mash1 and Pax6 (Figure 7A,B) with more nuclear staining evident in cells within the sustentacular cell layer of the ON at this time point (Figure 7E,F). Quantification of numbers of cells labeled with Mash1 and Pax6 was represented as the total number cells in the ON and not separately into specific cell types.

### X-ray irradiation of the nose disrupts olfactory function

To assess the olfactory function following X-ray irradiation of the mouse nose, two olfactory tests were performed: the Buried Food Pellet and the Habituation/Dishabituation test at 24 hours, 1 week, 2 weeks and 5 weeks (five animals were anlysed per group). The Buried Food Pellet measures the mouse's ability to find a buried piece of food using olfactory cues. The Habituation/Dishabituation test assesses the mouse’s ability to discriminate between different odours. A decrement in the amount of time the mouse spends with its nose to the odour is inferred to signal its ability to habituate to a repeatedly exposed odour. The increase in time exploring a new odour is interpreted to reflect dishabituation. Irradiated mice had no detectable change in behaviour with respect to controls either during their time in the home cage or during behavioural testing.

**Figure 8:**
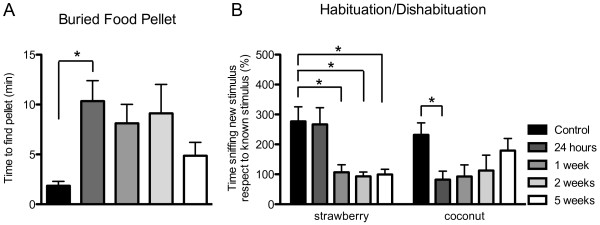
**Behavioural analysis of irradiated mice.A**, Buried Food Pellet test measures the latency to find a food pellet. A significant impairment was found 24 hours post X-ray irradiation (F_(4,34)_ = 2.754, p = 0.0437, n=5 mice). **B**, Habituation/Dishabituation test measures the amount of time spent in investigating a new odour, strawberry and coconut. A significant impairment was found 24 hours post X-ray irradiation in the identification of coconut, while for strawberry this impairment is only seen after 1 week (F_(4,27)_ = 2.540, p = 0.0629, n=5 mice; (F_(4,31)_ = 5.724, p = 0.0014), n=5 mice respectively). * p ≤ 0.05.

Results for the Buried Food Pellet test (Figure 8A) show a statistically significant difference between groups, as determined by One-way ANOVA. In particular, mice spent significantly more time to find the buried food pellet 24 hours post-irradiation, with respect to control group (p ≤ 0.05), indicating an impairment in the olfactory function at this time point. Moreover, there seems to be a trend to return to normal values, as seen 1, 2 and 5 weeks post-irradiation.

For the Habituation/Dishabituation test, we measured two different odours: strawberry and coconut (Figure 8B). For strawberry (with respect to water), we did not see a significant difference after 24 hours but only after 1 week (p ≤ 0.05), and this impairment was maintained up to 5 weeks. Instead, for coconut (with respect to strawberry), we saw a significant impairment in identifying this odour as new at 24 hours (p ≤ 0.05). Moreover, this impairment recovered over time, as seen after 1, 2 and 5 weeks. Overall, performances in both tests demonstrate a significant impairment in the olfactory function following X-ray irradiation of the nose.

## Discussion

Following X-ray irradiation, we observed an overall decrease in cell proliferation at 24 hours, a complete abolition of mitosis at 2 and 24 hours and a decrease in apoptosis at 2 hours. These data seem to indicate that the ON, and especially the active cycling zone of the ON, the basal lamina, has suffered a cell cycle arrest immediately after and as a consequence of ionizing radiation. X-ray irradiation has been shown before to induce permanent cell cycle arrest and the exhibition of senescence-like phenotypes in normal human cells [32] and to induce senescence in adult human mesenchymal stem cells [33]. We do not believe these cells have entered senescence, a process in which cells permanently stop dividing and that can happen after DNA damage following X-ray irradiation, because both proliferation and apoptosis tend to return to normal levels after 2–5 weeks. We believe these cells have entered a reversible cell cycle arrest triggered by ionizing radiation-induced DNA damage, from which they recover after only 1–2 weeks, confirming the plasticity of the ON. Following X-ray irradiation, there are variations in radiation sensitivity of neural stem and progenitor populations in the developing mouse brain [34,35]. This radiation sensitivity applies to cell proliferation and apoptosis. X-ray irradiation of the adult mouse nose did not cause increased cell death at any time points post-irradiation. 8 Gy of X-rays (used in this study) caused a significant increase in the numbers of apoptotic nuclei in the dentate subgranular zone following X-ray exposure to the brains of mice [36]. A single 1 Gy dose of irradiation caused an increase in apoptosis within the subventricular zone however, no apoptosis was observed in other brain regions [37]. Therefore different regions of the mouse central nervous system have varying levels of sensitivity to irradiation with respect to both the proliferation of neural precursors as well as cell death.

Contrary to other models of ON degeneration, which result in high morphological alterations and overall degeneration of the ON, our model seems to target specifically the proliferating cell populations, with minor morphological changes. We have found a disruption of the basal lamina at 1 and 2 weeks post-irradiation, but the overall gross morphology of the ON remained unaltered.

As expected, for all groups, the changes in proliferation and apoptosis mostly occurred in the basal lamina, indicating that this area is indeed actively cycling. This area is historically considered the locus for olfactory stem cells in the ON and the only zone for cell proliferation. Our results have also shown that apical sustentacular cells undergo proliferation and apoptosis. Following X-ray irradiation, there are greater numbers of cells in the apical layer of the ON expressing Ki67 compared to BrdU. It has been shown before that nestin, an intermediate filament protein expressed in progenitor cells of the neural lineage, in the ON is mainly expressed by endfeet and inferior processes of sustentacular cells [10]. It has also been shown that during mouse ON development, in particular at E12.5, there are two populations of dividing progenitors, located respectively on the apical and basal sides of the ON [38]. The proportion of basal progenitors then increases progressively, as development of the ON proceeds, and that of apical progenitors decreases [39,40]. It has been suggested that these apical progenitors, which express Mash1, would give rise to more mature progenitors that migrate basally [38]. Others have shown that apical progenitors also express the transcription factors Six1 and Hes1 [41] and that they may be progenitors for sustentacular cells. It could be that different genes regulate neurogenesis differently during embryonic and established neurogenesis [37,42,43]. The endfoot of the sustentacular cell is adjacent to the basal lamina of the ON. We have shown that the basal lamina is disrupted following X-ray irradiation. It is possible that this process causes the sustentacular cell to undergo morphological changes. A study of the human ON showed that nestin expression in sustentacular cells was related to olfactory function however, olfactory impairment was not connected with abnormalities at the basal cell level [44]. Overall, it seems that there might be indeed a role for sustentacular cells that is more complex that presently believed.

CD15-positive olfactory glandular cells migrate from the lamina propria into the basal layer of the ON corresponding to a severe disruption in the basal lamina at 1 and 2 weeks post-X-ray irradiation. A previous study showed that degeneration of the ON during normal aging spatially co-localizes with lesions in the Bowman's gland in the mouse olfactory mucosa [45]. We have observed activation/migration of CD15-positive glandular cells coinciding with an increase in olfactory neuron markers (carnosine and OMP), an increase in cell proliferation and an overall surge of activation/regeneration in the ON. CD15 is considered a marker for glandular cells but it has been shown previously to label adult neural stem cells [46,47]. The previous demonstration that olfactory cell populations marked by CD15 give rise to neurospheres [11], indicates that glandular cells marked by CD15 might have neurogenic potential. It has been proposed that there is a mesenchymal stem cell population in the lamina propria, which is able to cross to the ON [48,49]. The disruption of the basal lamina and the opportunity for CD15-positive glandular cell to migrate across the basal lamina as a consequence of X-ray irradiation, leads us to hypothesize that there may be a fluidic interaction between Bowman's gland in the lamina propria and the ON.

Mash1 is a transcription factor involved in neurogenic cell fate [50-52], and is necessary for the differentiation of OSNs in the ON [42]. Pax6 is a transcription factor expressed in embryonic and adult stem cells and involved in determination of neurogenic cell fate [53,54]. In the olfactory system Pax6 has been shown to be involved in the development of olfactory supporting cells [55]. Both Mash1 and Pax6 were expressed in cells throughout the ON (including sustentacular cells in the apical layer). The numbers of Pax6- and Mash1-positive nuclei are at their highest levels 24 hours post-irradiation and then decline from 1–5 weeks post-irradiation. This increase in transcription factors subsequently led to a greater number of mature OSNs 1 week post-irradiation. We know from previous experiments that an increase in the numbers of Mash1-positive cells followed a reduction in OSNs after bulbectomy [56]. Following methyl bromide lesion, expression of the transcription factor p63 is no longer evident in horizontal basal cells [9]. It is re-expressed 3 days post-lesion and by 7 days post-lesion, ORNs are evident [9]. However, another cue for regulating transcription factors is driven by the apoptosis of olfactory precursors. Over expression of the apoptosis signaling regulating kinase-1 (ASK1) in adult hippocampus-derived progenitor cells activated the Mash1 promoter [57].

X-ray irradiation has undoubtedly resulted in impairment in the olfactory function, measured by both the Buried Food Pellet test and the Habituation/Dishabituation test. It is evident at 24 hours post-irradiation that there is olfactory impairment. As olfactory bulbs were protected by a lead shield during irradiation, we do not think the olfactory impairment is due to a deficit in olfactory bulb function, OSN signaling via axons or odorant detection by olfactory neuron cilia. The examination of βIIITubulin expression in the cell soma, nerve fibres and cilia of OSNs in the ON and the nerve fibres, glomerular and mitral cells of the bulbs showed no change following irradiation. Previous studies have shown that βIIITubulin forms microtubules that form neurites [58] and that it is specific for the cilia, dendrites, somata and axons of OSNs [59,60]. Following axonal degeneration caused by bulbectomy in the rat, βIIITubulin expression is lost in OSNs [59]. This did not occur in our irradiated mice. In the Habituation/Dishabituation test one of the odours (strawberry) could still be identified after 24 hours and impairment for this odour was only seen 1 week post-irradiation. The repertoire of OSNs in the mouse ON is highly complex [61,62], so that the identification of each odour probably relies on the use of distinct or partially distinct OSN families, differently affected by our irradiation protocol. The Buried Food Pellet test relies on the animal’s natural tendency to use olfactory cues for foraging whereas the Habituation/Dishabituation test relies on the animal’s tendency to investigate novel smells and discriminate between different odours. The irradiation of the ON led to an immediate disruption of olfactory responses. Detection of general odorants by the vomeronasal organ has been reported [63,64]. Examination of the vomeronasal organs of the irradiated mice compared to the control group showed no histological aberrations. Therefore we do not attribute the loss of olfactory function to a defect in the vomeronasal organs of irradiated mice. However, we were able to show that the mucous layer as shown by CD15 expression was absent at 24 hours post-irradiation. Nagashima & Touhara [65] demonstrated that enzyme inhibitors in the mucosa cause the enzymatic conversion of odours fast enough to affect recognition of the odorant at the levels of the OSNs. Therefore, following X-ray irradiation the detection and discrimination of odours is affected by the differential presence of nasal mucous.

## Conclusions

This study has demonstrated that X-ray irradiation of the ON induces a reversible cell cycle arrest in the ON as well as a series of transcriptional alterations and a disruption of olfactory function which are mostly recovered 5 weeks post-irradiation. Coincident with changes to the basal lamina and basally situated olfactory stem cell populations, the sustentacular and glandular cells of the ON were shown for the first time to respond to irradiation by undergoing anatomical and spatial alterations. This study extends the understanding of ON plasticity and chemosensory function to include the role of olfactory supporting cells. Moreover, it might impact on the understanding of the effects on proliferating cells of ionizing radiation, which is commonly used in computed tomography scans, cancer radiotherapy and nuclear medicine.

## Methods

### Animals

All experiments were carried out using wild-type male C57BL/6 J mice at 8 weeks of age and have been approved by the Garvan Institute of Medical Research/St Vincent’s Hospital Animal Ethics Committee and conducted in accordance with the Australian code of practice for the care and use of animals for scientific purposes (7^th^ edition, 2004). Animals were kept in a 12-hour light/dark cycle, with food and water ad libitum and handled for 1 week before experimentation in order to diminish their stress or anxiety.

### X-ray irradiation

Mice were anaesthetized with an intraperitoneal injection of 75 mg/Kg Ketamine plus 15 mg/Kg Xylazine and placed in a circular Perspex pie chamber with 12 compartments. This was covered with a lead shield designed to protect the whole body except the nose snout (external diameter 21.5 cm, hole diameter 6 cm; Figure 1A). Care was taken to protect the olfactory bulbs. Mice were irradiated in groups of 5 with 8 Gy of X-rays, administered in a single dose at a rate of 0.9 Gy/min. X-ray irradiation was carried out in a X-RAD 320 X-ray irradiator (Precision X-Ray Inc.). Mice were randomly assigned to one of 5 groups with survival times of 2 hours (n=3 mice), 24 hours (n=8 mice), 1 week (n=8 mice), 2 weeks (n=8 mice) or 5 weeks (n=8 mice), with daily monitoring and weighing for the duration of the experiment. The control group was not irradiated and consisted of paired littermates.

### BrdU injection

The thymidine analogue 5-bromo-2'deoxyuridine (BrdU, Sigma) was diluted to 10 mg/ml in 0.9% saline solution and an intraperitoneal injection was administered at a dose of 50 mg/Kg body weight, 2 hours before transcardiac perfusion.

### Transcardiac perfusion and tissue processing

Mice were anaesthetized with an intraperitoneal injection of 100 mg/Kg Ketamine plus 20 mg/Kg Xylazine and perfused with heparinized saline (10 IU/ml) followed by 4% paraformaldehyde (PFA, Proscitech). Olfactory tissue was dissected together with the olfactory bulbs and post-fixed in 4% PFA overnight. The ON was decalcified for 7 days in 15% EDTA/4% PFA and processed for paraffin embedding. Coronal serial sections of 6 μm were collected via a microtome (Leica). Tissue sections were left at 60°C for 1 hour for melting of the paraffin, dewaxed in Histoclear solution (National Diagnostics) and rehydrated through a graded series of alcohol.

### Hematoxylin and eosin staining

H&E staining was performed for each animal, at intervals of 180 μm for the entire length of the mouse nose (including the OB and the vomeronasal organ), in order to determine the same coronal level for all animals analyzed. Sections were stained with hematoxylin for 45 seconds and washed in tap water and then stained with eosin for 30 seconds and washed in tap water. Tissue was dehydrated in a graded series of alcohols and slides mounted with Eukitt (Sigma).

### Immunohistochemistry

For immunohistochemical analysis against olfactory marker protein (OMP) and carnosine, antigen retrieval was performed by immersing slides in TE 1X pH 9.0 at high temperature. Non-specific staining was blocked in 10% goat and rabbit serum (Sigma) plus 1% bovine serum albumin for 1 h. Sections of ON and OB from control and irradiated mice were incubated with the primary antibody anti-βIIITubulin. The ON further examined using anti-OMP, anti-carnosine and (Table 1) overnight at 4°C. Control sections were incubated with 1% bovine serum albumin and processed in parallel. Endogenous peroxidase activity was quenched in 0.3% H_2_O_2_ in PBS for 15 min. Sections were incubated with the respective biotinylated goat anti-rabbit, horse anti-mouse or rabbit anti-goat secondary antibodies (1:300, Vector Laboratories) for 30 minutes at room temperature. Avidin-Horseradish Peroxidase complex was prepared using the Vector ABC Elite kit (Vector Laboratories) according to the manufacturer’s recommendations. Immunoreactivity was visualised using DAB as the chromogen and slides mounted using Aquamount (BDH, VWR).

**Table 1:** List of primary antibodies used for immunofluorescence and immunohistochemical analysis

**Antibodies**	**Cell type**	**Origin**	**Company**	**Dilution**	**Antibody pre-treatment**	**Abbreviation**
anti-ASH1 (MASH1)	neurogenic precursors	rabbit polyclonal	Millipore	1:20	Citrate buffer	Mash1
anti-bromodeoxyuridine	proliferative cells	mouse monoclonal	Millipore	1:50	HCl	BrdU
anti-carnosine	immature and mature OSNs	rabbit polyclonal	Millipore	1:50	TE buffer	carnosine
anti-CD15	glandular cells	mouse monoclonal	BD Biosciences	1:10	Citrate buffer	CD15
anti-glial fibrillary acidic protein	olfactory ensheathing glia	rabbit polyclonal	Dako	1:250	nil	GFAP
anti-keratin	HBCs	rabbit polyclonal	Dako	1:200	nil	keratin
anti-Ki-67, clone SP6	proliferative cells	rabbit monoclonal	Thermo Scientific	1:50	nil	Ki67
anti-olfactory marker protein	mature OSNs	goat polyclonal	Santa Cruz Biotechnology	1:800	TE buffer	OMP
anti-Pax6, C-terminus	neurogenic precursors	rabbit polyclonal	Millipore	1:200	Citrate buffer	Pax6
anti-phospho-Histone H3 [pSer^10^]	mitotic marker	rabbit polyclonal	Sigma	1:200	nil	pH3
anti-βIII Tubulin	immature and mature OSNs	mouse monoclonal	Promega	1:800	nil	βIIITubulin

### Immunofluorescence analysis

A list of all primary antibodies used for immunofluorescence experiments can be found in Table 1. Antigen retrieval with citrate buffer was performed by immersing slides in 8.2 mM tri-sodium citrate plus 1.8 mM citric acid at high temperature. HCl treatment was performed by incubating sections in 2 M HCl at 60°C for 35 minutes. Blocking was performed with 10% goat serum (Sigma) and tissue permeabilization with 0.1% (v/v) Triton X-100, except for anti-Ki67, anti-BrdU and anti-pH3, where 0.3% (v/v) Triton X-100 was used. Sections were incubated with the primary antibody overnight at 4°C. Primary antibodies were probed with the secondary antibodies Alexa Fluor® 488 goat anti-rabbit or goat anti-mouse (1:500, Molecular Probes, Invitrogen) or Cy3 goat anti-rabbit or goat anti-mouse (1:1000, Jackson ImmunoResearch) for 45 minutes at room temperature. Cell nuclei were stained with DAPI (Molecular Probes, Invitrogen) and slides mounted using FluorSave (Calbiochem).

For immunofluorescence double labeling, the following antibody combinations were adopted: anti-BrdU plus anti-keratin, anti-CD15 plus anti-keratin, anti-GFAP plus anti-βIIITubulin.

### TUNEL assay

The TUNEL assay was performed by use of the DeadEnd^TM^ Fluorometric TUNEL System (Promega), according to manufacturer's instructions. Briefly, tissue was hydrated and permeabilized by Proteinase K (20 μg/ml) digestion for 8 minutes at room temperature. No further PFA fixation was performed. Tissue was incubated for 1 hour at 37°C with Terminal Deoxynucleotidyl Transferase (recombinant) and fluorescein-12-dUTP and reaction stopped by incubating slides in 2x SSC for 15 minutes. Cell nuclei counterstaining was performed by use of DAPI (Molecular Probes, Invitrogen).

### Imaging and quantification

For all immunofluorescence analysis, sections were examined using an upright fluorescence microscope (Axioplan, Carl Zeiss) and pictures were acquired using a 40X and 100X oil objectives and an AxioCam digital camera (Carl Zeiss MicroImaging GmbH). For all histochemical analysis, sections were examined using an upright microscope (Axiophot, Carl Zeiss MicroImaging GmbH) and pictures acquired using a 2.5X and 40X dry objectives and an AxioCam digital camera (Carl Zeiss MicroImaging GmbH). No image manipulation has been performed of any kind, apart from rotation and cropping.

For each animal and marker, 5 different areas of the ON in the coronal section of the nose were quantified by counting the number of positively stained cells, corresponding to the middle of the nasal septum, one more superior and one more inferior area of both the left and right turbinates (Figure 1B). For all sections analyzed, these areas remained constant and were examined at the level of 100–250 μm anteriorly, with respect to the end of the olfactory bulbs. For each area quantified, 200 μm length of ON was analyzed (Figure 1C). The mean number of positive cells between areas was determined for each section and these values used to determine the mean number of positive cells between animals (n=3-5).

### Olfactory behavioural tests

Olfactory tests have been performed as previously described [11], with minor alterations. Mice were paired littermates to avoid possible differences caused by the animal’s age. All animals performed each test only once.

### Buried food pellet

This test measures the mouse’s ability to find a buried food pellet using olfactory cues. Mice (n=5 per group) were food deprived overnight with free access to water, between 5 pm the day prior to the test and the beginning of the test at 8 am the following day. To avoid problems associated with neophobia, mice were habituated to eat the new food by placing pieces in the home cage overnight for 3 days before the test. On the day of the test, a sultana was placed underneath the bedding material within the home cage. The floor of the cage was then completely covered with new bedding material to a depth of approximately 2.5 cm. The latency to find the sultana and start eating was recorded using a stopwatch. A maximum cut-off time of 15 minutes was used and mice reaching the cut-off were included in the analysis as if they had found the pellet after 15 minutes.

### Habituation/Dishabituation

Odorants were prepared by diluting strawberry and coconut essences (Queen Fine Foods Pty Ltd) 1:100 in distilled water. Mice were placed individually into a clean cage. Cotton buds were dipped into distilled water or odorant and are then placed through the wire cage lid and fixed with the tip at a height of 5 cm above the cage floor. Each stimulus was presented 3 times consecutively in the following order: water 3x, strawberry 3x, coconut 3x, for 3 minutes each and with the mouse returning to his home cage for 1 minute between each stimulus presentation. The cumulative time spent sniffing each stimulus was measured. The behaviour sniffing is defined as orienting the nose towards the stimulus within 2 cm of the cotton tip or in contact with the tip. Chewing the cotton tip or contacting it with an open mouth is not counted as sniffing. The percentage of time spent sniffing the first strawberry presentation with respect to the third water was calculated. The percentage of the time spent sniffing the first coconut presentation with respect to the third strawberry was calculated.

### Statistical analysis

All results were expressed as MEAN ± SEM plotted on graph and statistical analysis was performed using GraphPad Prism software (version 5.0), with statistical significance set at p ≤ 0.05. For both olfactory tests, a one-way ANOVA was performed, followed by Dunnet’s post hoc test, with respect to control group. For all immunohistochemistry analysis, a one-way ANOVA was performed, followed by Tukey's post hoc test, for comparison between all groups.

## Abbreviations

BrdU: 5-bromo-2’deoxyuridine; GFAP: glial fibrillary associated protein; OB: olfactory bulb; OMP: olfactory marker protein; ON: olfactory neuroepithelium; OSN: olfactory sensory neuron; PBS: phosphate buffered saline; PFA: paraformaldehyde; TUNEL: terminal deoxynucleotidyl transferase mediated UTP nick end labeling.

## Competing interests

The authors declare that they have no competing interests.

## Authors’ contributions

CC designed the study, carried out the immunofluorescence and animal studies, performed the statistical analysis and wrote the manuscript. YH carried out the immunohistochemistry studies. JS participated in the design of the study. KD conceived the study, participated in its design and coordination and helped to draft the manuscript. All authors read and approved the final manuscript.

## Supplementary Material

Additional file 1 High magnification photomicrographs of βIIITubulin labeling of the olfactory bulbs in control and irradiated mice. Click here for file
