# Supplementary material for: Morphological and behavioural changes occur following the X-ray irradiation of the adult mouse olfactory neuroepithelium
Source: BMC Neurosci. 2012 Oct 31;13:134. doi: 10.1186/1471-2202-13-134 (PMC3536589; doi:10.1186/1471-2202-13-134)
Supplement: Additional file 1 — High magnification photomicrographs of βIIITubulin labeling of the olfactory bulbs in control and irradiated mice. [file 1471-2202-13-134-S1.pdf]

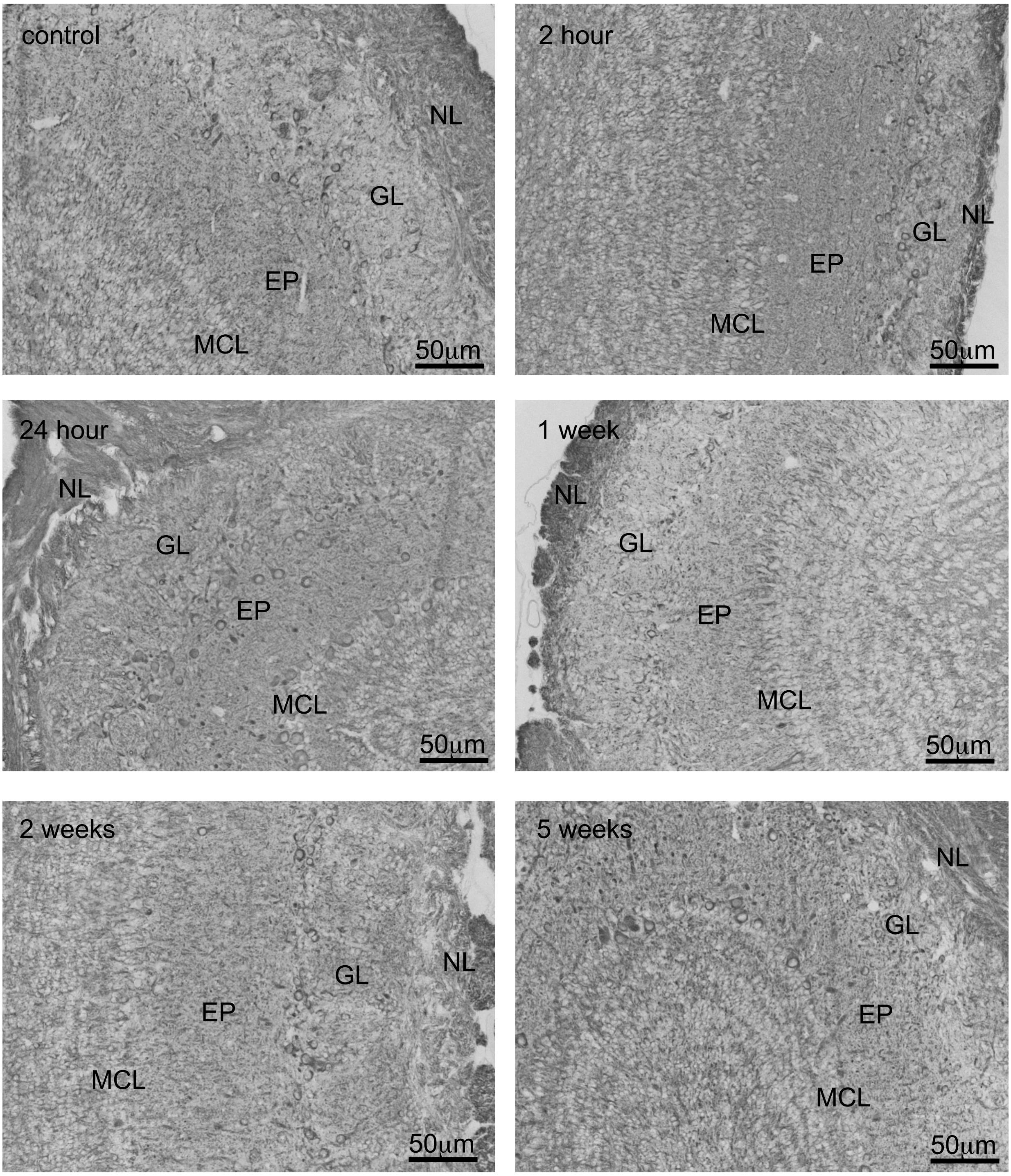

Additional Figure 1: Photomicrographs of mouse olfactory bulbs in control and irradiated mice labelled with βIIITubulin. In all mice examined (n=3 mice per group) there was no change in expression levels of βIIITubulin. There was also no morphological alterations post-irradiation. (NL=nerve fibre layer, GL=glomerular layer, EP=external plexiform layer, MCL=mitral cell layer)
